# Vaccination with Live or Heat-Killed Aspergillus fumigatus Δ*sglA* Conidia Fully Protects Immunocompromised Mice from Invasive Aspergillosis

**DOI:** 10.1128/mbio.02328-22

**Published:** 2022-09-06

**Authors:** Caroline Mota Fernandes, Tyler G. Normile, Joao Henrique Tadini Marilhano Fabri, Veronica Soares Brauer, Glauber R. de S. Araújo, Susana Frases, Leonardo Nimrichter, Iran Malavazi, Maurizio Del Poeta

**Affiliations:** a Department of Microbiology and Immunology, Stony Brook University, Stony Brook, New York, USA; b Departamento de Genética e Evolução, Centro de Ciências Biológicas e da Saúde, Universidade Federal de São Carlos, São Carlos, Brazil; c Instituto de Biofisica Carlos Chagas Filho, Universidade Federal do Rio de Janeiro, Rio de Janeiro, Brazil; d Instituto de Microbiologia Paulo de Góes, Universidade Federal do Rio de Janeiro, Rio de Janeiro, Brazil; e Division of Infectious Diseases, School of Medicine, Stony Brook University, Stony Brook, New York, USA; f Veterans Administration Medical Center, Northport, New York, USA; University of Florida

**Keywords:** fungal infection, *Aspergillus*, invasive aspergillosis, vaccine, sterylglucosides, immunocompromised host, conidia, *Aspergillus fumigatus*, immunization

## Abstract

Aspergillus fumigatus causes invasive aspergillosis (IA) in immunocompromised patients, resulting in high mortality rates. Currently, no vaccine formulations to promote immune protection in at-risk individuals have been developed. In this work, we deleted the sterylglucosidase-encoding gene, *sglA*, in Aspergillus fumigatus and investigated its role in fungal virulence and host vaccine protection. The Δ*sglA* mutant accumulated sterylglucosides (SGs), newly studied immunomodulatory glycolipids, and exhibited reduced hyphal growth and altered compositions of cell wall polysaccharides. Interestingly, the Δ*sglA* mutant was avirulent in two murine models of IA and was fully eliminated from the lungs. Both corticosteroid-induced immunosuppressed and cyclophosphamide-induced leukopenic mice vaccinated with live or heat-killed Δ*sglA* conidia were fully protected against a lethal wild-type A. fumigatus challenge. These results highlight the potential of SG-accumulating strains as safe and promising vaccine formulations against invasive fungal infections.

## INTRODUCTION

Invasive fungal infections account for ~1.5 million deaths a year and mainly affect immunocompromised individuals, including AIDS patients, solid-organ transplant recipients, and those undergoing immunosuppressive therapies (e.g., immunosuppressive drugs or chemotherapy) (1–3). Among these fungal infections, invasive aspergillosis (IA) caused by Aspergillus fumigatus afflicts more than 200,000 people worldwide each year, with a mortality rate ranging from 30% to 95% (4). Factors such as the host immune status at the time of exposure and the nature of immunosuppression (neutropenia compared to corticosteroid induced) influence the outcome of IA, which exhibits high mortality rates despite the availability of mold-active antifungal drugs (4–6).

A. fumigatus is a saprotrophic fungus ubiquitously found in the environment that causes both acute and chronic illnesses in at-risk individuals (7). The primary route of exposure to A. fumigatus begins with the production of conidia, or asexual spores, which become dispersed in the air and subsequently are inhaled into the lungs (6). In fact, it is estimated that humans inhale hundreds of A. fumigatus conidia every day, which readily reach the alveolar spaces due to their relatively small size (2 to 3 μm), indicating a constant daily battle at the host-pathogen interface in the upper respiratory tract and lower airways (8, 9). Healthy individuals exposed to A. fumigatus mount an appropriate immune response, resulting in the pulmonary clearance of the fungus (10). However, individuals with compromised immune systems fail to control the inhaled conidia, which germinate to hyphae upon entering the lung parenchyma, potentially causing invasive disease. In addition, injury to the respiratory tract caused by, for example, severe acute respiratory syndrome coronavirus 2 (SARS-CoV-2) in patients with respiratory distress syndrome (11) stimulates fungal invasion and the emergence of coronavirus disease 2019 (COVID-19)-associated pulmonary aspergillosis (12, 13).

Azoles constitute the main antifungal class used for the clinical management of IA (14). These drugs target the enzyme lanosterol 14α-demethylase, impairing the synthesis of the most abundant fungal sterol, ergosterol. However, several A. fumigatus isolates that are resistant to azoles have been identified to date, leading to a significant reduction in the efficacy of antifungal treatment. No fungal vaccines are currently available for clinical use, most notably due to the challenge of inducing protective immunity in immunocompromised hosts. Thus, there is a dire need for safe and effective vaccine formulations to combat fungal infections, including A. fumigatus infections, in at-risk hosts.

Although there has been research into the development of vaccines against A. fumigatus, efficacy has been lacking because the cell types lost during immunosuppression are the ones responsible for host protection (15–18). Several studies in the literature have attempted to use specific fungal components or whole-cell Aspergillus conidia/hyphae as strategies to induce protective host immunity (5, 19). For example, β-glucans are constituents of the fungal cell wall that induce complement activation in the host, ultimately leading to fungal opsonization via the C-type lectin receptor dectin-1 (20–22). Vaccine preparations containing β-glucans have been previously well tolerated in clinical trials, including among patients undergoing chemotherapy (23). The potential of β-1,3-glucans to induce a protective response against Aspergillus was also corroborated by vaccinating mice with heat-killed (HK) Saccharomyces cerevisiae (24). Torosantucci and colleagues utilized a glycoconjugate vaccine (laminarin) by fusing β-glucan to diphtheria toxoid, a vaccine adjuvant, which elicits antibody production (25). Mice immunized with laminarin exhibited significantly prolonged survival against a lethal challenge with A. fumigatus, highlighting the potential of β-glucans as antifungal vaccines. Besides individual cell wall constituents, vaccination with an A. fumigatus hypha sonicate preparation protected 70% of immunocompromised mice from developing lethal aspergillosis (26, 27). More recently, it was shown that a heat-killed Cryptococcus Δ*fbp1* strain, lacking a subunit of the SCF E3 ligase, induces protection against invasive aspergillosis (28). Despite the breadth of work in these studies and others, no vaccines have yet reached the efficacy and/or safety profile needed to be considered for clinical trials against aspergillosis.

We previously reported that Cryptococcus neoformans Δ*sgl1*, a mutant strain that accumulates sterylglucosides (SGs) due to the deletion of the sterylglucosidase-encoding gene (*SGL1*), was avirulent in a mouse model of infection and conferred complete host protection against a subsequent lethal challenge with the wild type (WT) in several models of immunosuppression (29, 30). SGs are immunomodulatory glycolipids with a wide array of applications (reviewed in reference 31) but were only recently shown to protect immunocompromised mice from lethal infection with C. neoformans, possibly functioning as potent adjuvants for fungal antigens (29, 32, 33). Moreover, *in vivo* treatments with Sgl1 inhibitors restricted WT C. neoformans infection to the lungs (34). Interestingly, these inhibitors restricted WT cell growth under low-oxygen conditions, which was similar to the phenotype observed with C. neoformans Δ*sgl1*.

In the present study, we deleted the sterylglucosidase-encoding gene (*sglA*) in A. fumigatus and investigated the effects of this mutation on fungal virulence and host vaccine protection. We found that A. fumigatus Δ*sglA* exhibited increased levels of ergosteryl-3β-glucoside (fungally derived SGs), especially after 12 h of growth. The peak in SG accumulation was followed by reduced hyphal growth, suggesting that an excess of glycosylated sterols in the plasma membrane may delay the establishment of the cell polarity axis in A. fumigatus. Furthermore, the A. fumigatus Δ*sglA* mycelium produced a dense extracellular matrix (ECM) possibly composed of cell wall glycoconjugates that were enriched in the mutant compared to the WT and complemented strains. *In vivo*, A. fumigatus Δ*sglA* exhibited impaired virulence during primary infection, being fully cleared from the lungs of immunocompromised mice. Amazingly, immunosuppressed or neutropenic mice that were previously administered either live or HK A. fumigatus Δ*sglA* conidia were completely protected against a subsequent lethal A. fumigatus challenge. The immune protection was specifically triggered by the SG-accumulating mutant since mice administered HK WT conidia displayed a mortality rate similar to that in the unvaccinated group (phosphate-buffered saline [PBS]-administered mice). Taken together, our results strongly suggest that fungally derived SGs constitute a promising immunomodulatory compound for the further development of a highly efficacious vaccine for immunocompromised patients. To our knowledge, this is the first vaccine formulation that provides complete protection against A. fumigatus infection under immunocompromised conditions for hosts who are the most at risk for the development of IA.

## RESULTS

### The *sglA* gene encodes A. fumigatus sterylglucosidase.

The C. neoformans gene (CNAG_05607) encoding the sterylglucosidase enzyme has been identified previously (30, 35). Bioinformatic analysis of the A. fumigatus genome using CNAG_05697 as a query revealed that the sequence encoded by Afua_3g08820 constitutes a putative sterylglucosidase (E value of 0.0; 43% identity and 58% similarity), and this sequence is referred to as *sglA* in this study. Additionally, the alignment of the SglA amino acid sequence with those of other fungal homologs was described previously (34).

To characterize the relevance of *sglA* to A. fumigatus biology, we generated a mutant strain in which the *sglA* open reading frame (ORF) was replaced by the *pyrG* gene. Briefly, a deletion cassette containing *pyrG* flanked by 2 kb of the 5′ and 3′ untranslated regions (UTRs) was inserted into WT protoplasts (see Fig. S1 in the supplemental material), and transformants were selected based on their ability to grow in the absence of uracil and uridine (36, 37). To reconstitute *sglA*, Δ*sglA* protoplasts were transformed with a construct containing the *sglA* ORF fused to the *hph* gene, a marker that confers resistance to hygromycin B (38) (Fig. S1). Similarly to the gene deletion process, *sglA* reconstitution was guided by the 2-kb homology arms containing the 5′- and 3′-UTR sequences (37). Southern blot analysis using a 5′-UTR probe revealed the presence of a 1.8-kb fragment corresponding to the WT and reconstituted (Δ*sglA*::*sglA*^+^) constructs, while the expected 0.9-kb band was observed for the Δ*sglA* mutant (Fig. S1), confirming that the deletion and reconstitution events occurred at the *sglA* locus.

10.1128/mbio.02328-22.1 FIG S1 Deletion and reconstitution of the A. fumigatus *sglA* gene. Shown is a schematic representation of the strategy to confirm the deletion and reconstitution of the A. fumigatus *sglA* gene. A total of 650 bp of the 5′ UTR was amplified from WT genomic DNA, labeled with [α-^32^P]dCTP, and used as a radioactive probe. Digestion of the WT and Δ*sglA*::*sglA*^+^ genomic DNAs with HindIII generated a 1.8-kb fragment, in contrast to the 900-bp band observed for the Δ*sglA* strain in the blot on the right. Download FIG S1, TIF file, 0.9 MB. Copyright © 2022 Fernandes et al. 2022 Fernandes et al. https://creativecommons.org/licenses/by/4.0/ This content is distributed under the terms of the Creative Commons Attribution 4.0 International license.

**FIG 1:**
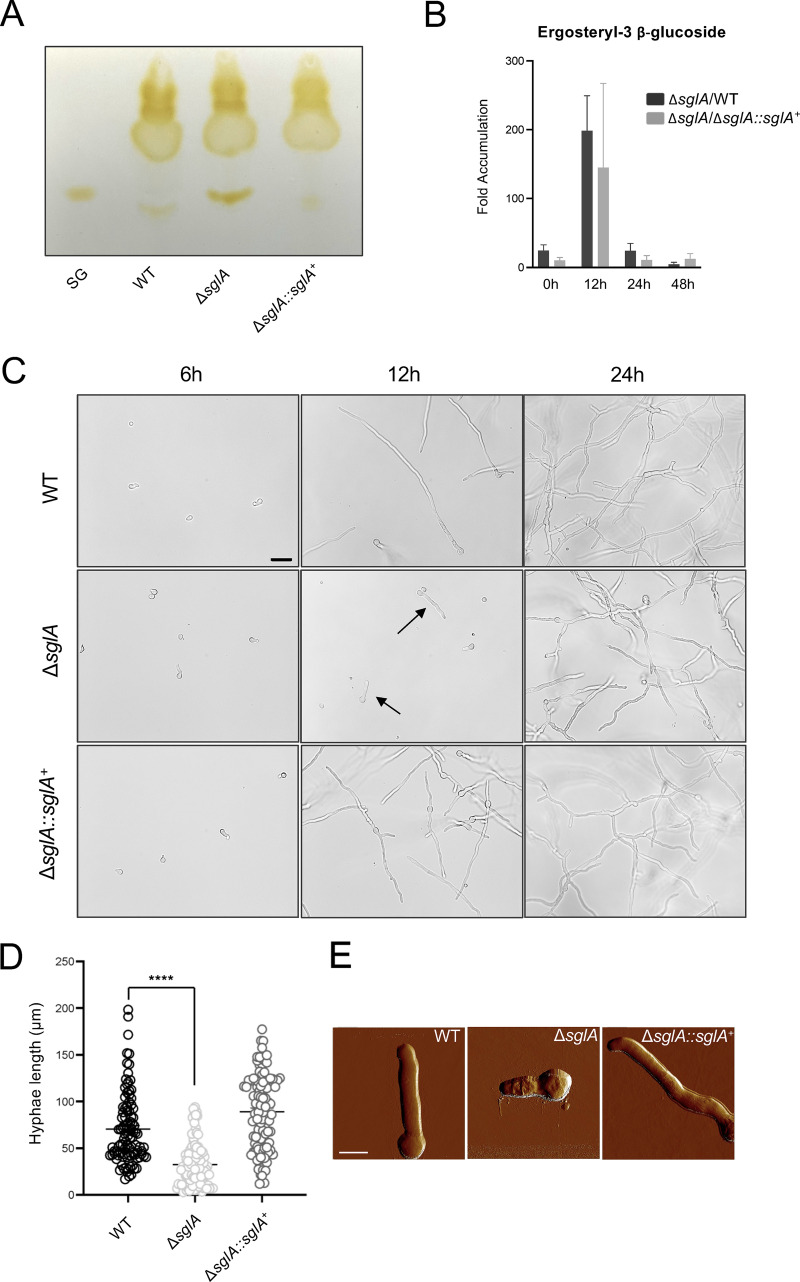
*sglA* is required for the hyphal growth and surface topography of A. fumigatus. (A and B) Analysis of the SG content was performed by thin-layer chromatography qualitatively comparing the bands to a known SG standard as the control (A) and by mass spectrometry quantitatively analyzing the levels of ergosteryl-3β-glucosides (fungal SGs), represented as fold changes for the Δ*sglA* strain compared to the WT or reconstituted strains (B). (C) WT, Δ*sglA*, and Δ*sglA*::*sglA*^+^ conidia were inoculated into liquid medium and grown at 37°C for 6, 12, and 24 h. Bar, 20 μm. Arrowheads indicate the reduced size of Δ*sglA* germlings in comparison to WT and Δ*sglA*::*sglA*^+^ hyphae. (D) Quantification of the average hypha sizes after 12 h of growth (*n* = 100 hyphae for each strain). Graphed data represent the means ± standard errors of the means (SEM). Significance was determined by one-way analysis of variance (ANOVA) using Dunnett’s multiple-comparison test for *P* value adjustment (****, *P < *0.001 for the Δ*sglA* mutant versus the WT). (E) Atomic force microscopy (AFM) images of the WT, Δ*sglA*, and Δ*sglA*::*sglA*^+^ strains in peak force mode. The scanned area was 25 μm. Bar, 5 μm.

Since sterylglucosidases act in SG catabolism, a strain lacking this enzyme should exhibit higher SG levels than those of the WT and reconstituted strains, so we investigated the levels of SGs in the Δ*sglA* mutant strain. Indeed, A. fumigatus Δ*sglA* displayed a drastic accumulation of SGs compared to the WT or the reconstituted strain (Fig. 1A). To better understand SG metabolism during different stages of the A. fumigatus life cycle, the SG content was quantified by mass spectrometry (MS) after conidia were harvested from a solid plate (0 h) or grown for 12, 24, and 48 h at 37°C in liquid minimal medium (MM). Interestingly, A. fumigatus conidia exhibited a higher SG content than those of hyphae grown for up to 48 h (Table S1). Also, the ratio of SGs in the Δ*sglA* mutant was dramatically higher than that of the WT (Δ*sglA*/WT) or the reconstituted strain (Δ*sglA*/Δ*sglA*::*sglA*^+^) at all time points, especially after 12 h (Fig. 1B).

10.1128/mbio.02328-22.5 TABLE S1 SG (ergosteryl-3β-glucoside) contents after WT, Δ*sglA*, and Δ*sglA*::*sglA*^+^ conidia were harvested from the solid plate (0 h) or grown for 12, 24, and 48 h at 37°C in liquid minimal medium. Download Table S1, TIF file, 0.01 MB. Copyright © 2022 Fernandes et al. 2022 Fernandes et al. https://creativecommons.org/licenses/by/4.0/ This content is distributed under the terms of the Creative Commons Attribution 4.0 International license.

### Disruption of the *sglA* gene delays early hyphal growth in A. fumigatus.

Because the greatest fold change difference in the levels of SGs was observed after 12 h of growth, this time point was selected for all downstream phenotypic analyses. Both the WT and reconstituted strains exhibited regular growth patterns, with germlings developing into hyphae at between 6 and 12 h at 37°C (Fig. 1C). In contrast, A. fumigatus Δ*sglA* exhibited shorter germlings and delayed growth, especially at 12 h (Fig. 1C, black arrows). Quantification of the length of hyphae revealed that the WT and Δ*sglA*::*sglA*^+^ strains had average lengths of 70.6 and 89 μm, respectively, while A. fumigatus Δ*sglA* germlings displayed an average length of 30.2 μm (Fig. 1D). In addition, the Δ*sglA* mutant exhibited a distinct surface topography compared to those of the WT and reconstituted strains (Fig. 1E). These results suggest that the accumulation of SGs alters the hyphal structure and hinders hyphal elongation.

### A. fumigatus Δ*sglA* exhibits reduced adhesion and a distinct cell wall architecture.

**FIG 2:**
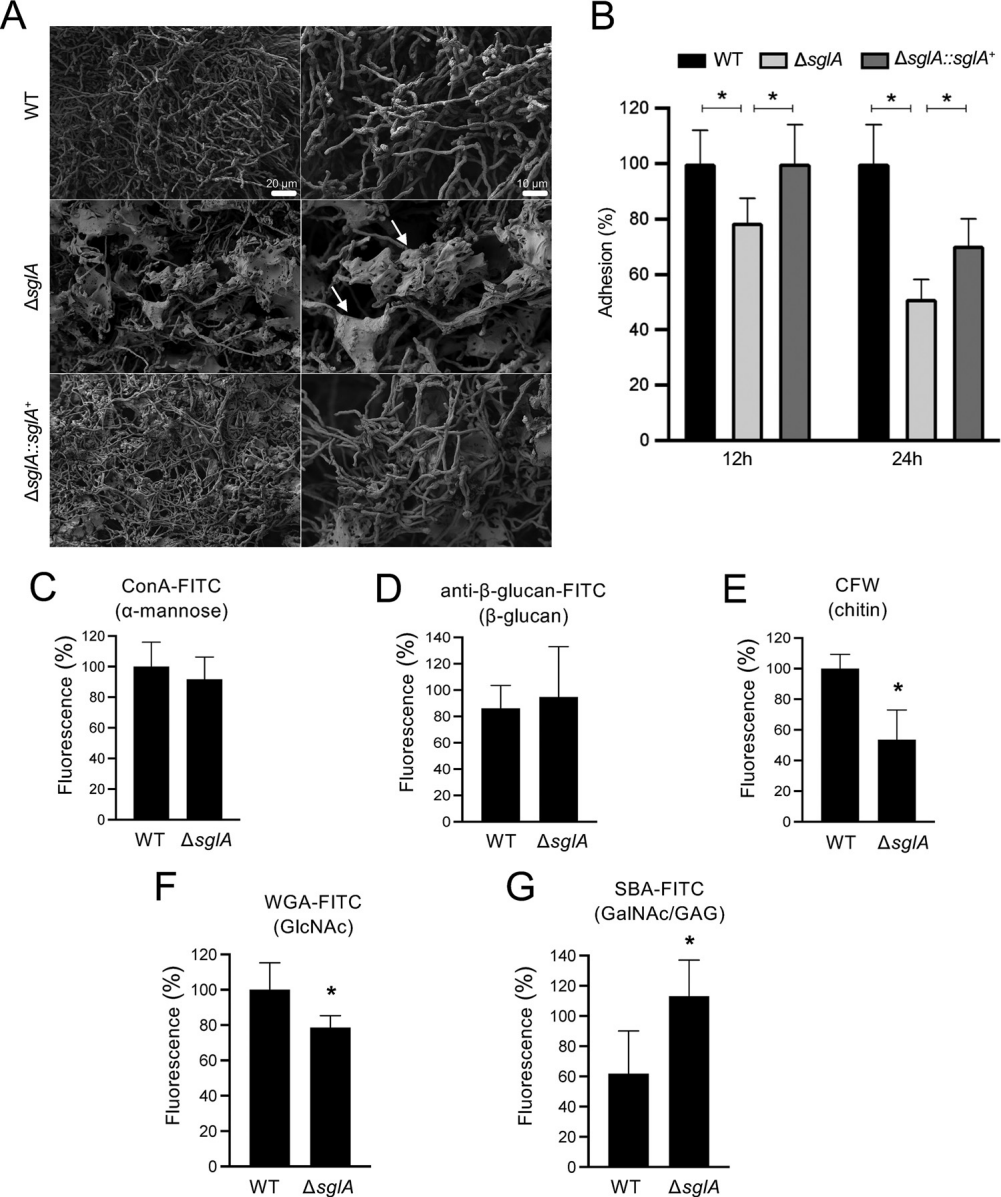
A. fumigatus Δ*sglA* exhibits a dense extracellular matrix, reduced adhesion, and altered production of cell wall polysaccharides. (A) Scanning electron microscopy of the WT, Δ*sglA*, and Δ*sglA*::*sglA*^+^ strains grown for 12 h at 37°C. Arrows indicate the presence of a dense extracellular matrix in the Δ*sglA* mutant. (B) Biofilm formation was evaluated by crystal violet absorbance at 570 nm and is expressed as percent adhesion considering 100% adhesion for the WT strain. Experiments were performed in 12 replicates, and the graphed data represent the means ± standard deviations (SD) (*, *P < *0.001). (C to G) Evaluation of specific carbohydrate contents exposed on the cell surface of the WT or Δ*sglA* strain. Cell surface α-mannose (C), β-glucan (D), chitin (E), *N*-acetylglucosamine (GlcNAc) (F), and *N*-acetylgalactosamine (GalNAc) (G) contents were measured after the strains were grown in minimal medium for 16 h. Experiments were performed in at least six replicates, and the graphed data represent the percentages of the ratios of the specific fluorescence signals to the fluorescence of resazurin, which was used to normalize the growth of the strains. Significance was determined via one-way ANOVA comparing the Δ*sglA* mutant to the WT strain (*, *P < *0.05).

In C. neoformans, the deletion of the *SGL1* gene resulted in distinct alterations to the capsule structure (32). C. neoformans Δ*sgl1* produced a thicker capsule than the WT or Δ*sgl1*+*SGL1* strain (Fig. S2), which suggested that the increased SG content influences the architecture of the glucuronoxylomannan (GXM)-rich capsule. Since A. fumigatus usually grows as a biofilm with hyphae embedded in a polysaccharide-rich extracellular matrix (ECM) (39, 40), we therefore investigated if the increased levels of SGs perturbed the polysaccharide organization in A. fumigatus. To do this, we used scanning electron microscopy (SEM) to analyze the cell wall structures of the WT, Δ*sglA*, and Δ*sglA*::*sglA*^+^ strains (Fig. 2A and Fig. S3). While the parental WT strain exhibited smooth hyphae, a dense ECM was seen in the A. fumigatus Δ*sglA* mycelium (Fig. 2A and Fig. S3, white arrows). However, the WT phenotype was only partially restored in the reconstituted strain. The electron micrographs display a thick ECM in the Δ*sglA*::*sglA*^+^ biofilm, although this is much less abundant than for the Δ*sglA* mutant (Fig. 2A and Fig. S3).

10.1128/mbio.02328-22.2 FIG S2 Scanning electron microscopy displaying the polysaccharide capsules of the WT, Δ*sgl1*, and Δ*sgl1*+*SGL1* strains of C. neoformans. Bars, 1 μm. Download FIG S2, TIF file, 1.7 MB. Copyright © 2022 Fernandes et al. 2022 Fernandes et al. https://creativecommons.org/licenses/by/4.0/ This content is distributed under the terms of the Creative Commons Attribution 4.0 International license.

10.1128/mbio.02328-22.3 FIG S3 Scanning electron microscopy of the WT, Δ*sglA*, and Δ*sglA*::*sglA*^+^ strains of A. fumigatus. Bars, 20 μm (left) and 10 μm (right). Arrows highlight the presence of a dense extracellular matrix in the Δ*sglA* mutant. Download FIG S3, TIF file, 1.2 MB. Copyright © 2022 Fernandes et al. 2022 Fernandes et al. https://creativecommons.org/licenses/by/4.0/ This content is distributed under the terms of the Creative Commons Attribution 4.0 International license.

Previous studies have shown that the ECM is required for A. fumigatus adhesion and virulence (41, 42). Therefore, we asked whether A. fumigatus Δ*sglA*, which has now been shown to contain a dense ECM, would exhibit increased rates of biofilm formation and surface adherence compared to those of the WT strain. Surprisingly, A. fumigatus Δ*sglA* exhibited significantly impaired biofilm formation after 12 and 24 h of growth compared to the WT and reconstituted strains (Fig. 2B). These results suggest that the dense ECM in A. fumigatus Δ*sglA* is dysfunctional and reduces its ability to adhere to surfaces. This is most likely due to the unbalanced proportion of polysaccharides that abnormally accumulate in the modified ECM in the Δ*sglA* mutant strain.

Thus, to explore the effect of SG accumulation on the cell wall carbohydrate composition, the WT, Δ*sglA*, and Δ*sglA*::*sglA*^+^ strains were stained with specific cell wall dyes, fluorescently labeled lectins, and antibodies to examine the exposed carbohydrates on the cell surface. We utilized (i) concanavalin A (ConA)-fluorescein isothiocyanate (FITC), which binds to mannose; (ii) anti-β-glucan–FITC, which recognizes β-glucans; (iii) calcofluor white (CFW), which labels chitins (polymers of β-1,4-GalNAc [*N*-acetylgalactosamine]); (iv) wheat germ agglutinin (WGA)-FITC, which recognizes exposed *N*-acetylglucosamine (GlcNAc) residues; and (v) soybean agglutinin (SBA)-FITC, which binds to GalNAc and galactose residues of the galactosaminogalactan (GAG). While no significant differences in the exposure of mannose residues (Fig. 2C) or β-glucans (Fig. 2D) were observed between the WT and Δ*sglA* strains, A. fumigatus Δ*sglA* displayed significantly decreased surface-exposed chitin (Fig. 2E) and GlcNAc (Fig. 2F) as well as a significant, nearly 2-fold increase in GalNAc/GAG (Fig. 2G) compared to the WT strain. GAG levels have been previously reported to directly impact the ECM composition of the A. fumigatus cell wall (42); therefore, the increased GAG level in A. fumigatus Δ*sglA* may correlate with the abundant ECM density in these hyphae (Fig. 2A). Together, these results suggest that increased levels of SGs interfere with the surface exposure of certain cell wall polysaccharides, the composition of the ECM, and, ultimately, the cell wall surface structure.

### A. fumigatus Δ*sglA* is avirulent in a mouse model of infection.

**FIG 3:**
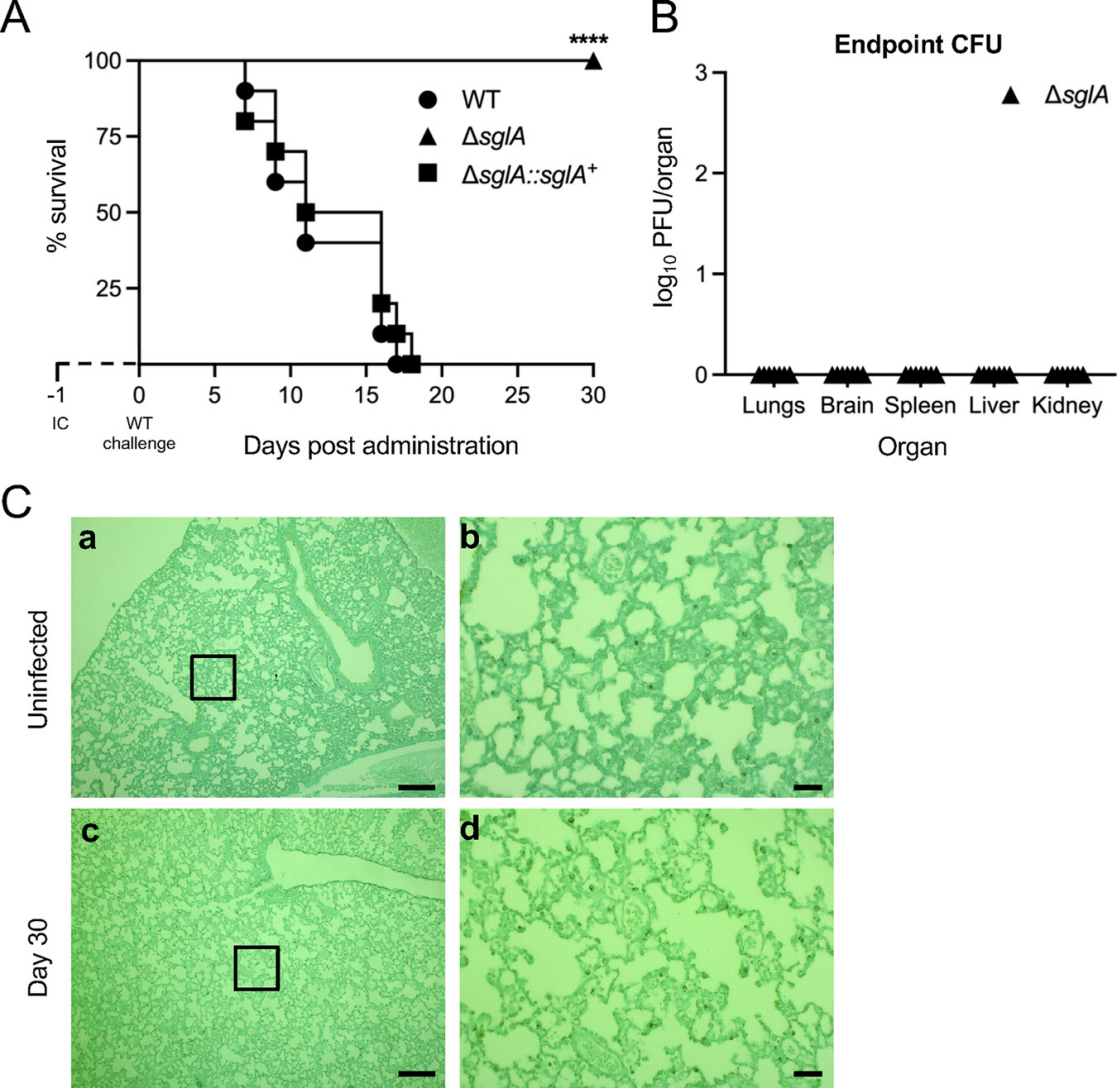
A. fumigatus Δ*sglA* is avirulent *in vivo*. (A) CBA/J mice (*n* = 10 mice/group) were immunocompromised (IC) via the subcutaneous administration of 1 mg/mouse triamcinolone acetonide 24 h prior to the intranasal instillation of either the WT, Δ*sglA*, or Δ*sglA*::*sglA*^+^ strain and assessed for survival over 30 days. (B) Mice that survived the 30-day infection were sacrificed, and the organ fungal burden was quantified (*n* = 7 mice). (C) Representative lung histopathology from an uninfected mouse and a mouse 30 days after inoculation with A. fumigatus Δ*sglA* conidia (*n* = 3 mice/group). Bars = 200 μm (a and c) and 50 μm (b and d). Graphed data represent the percent survival (A) and the means ± SD (B). Statistical significance was determined by the Mantel-Cox log rank test (A). Significance is indicated in panel A (****, *P < *0.001 for the Δ*sglA* strain versus either the WT or the Δ*sglA*::*sglA*^+^ strain).

We have previously shown that C. neoformans Δ*sgl1* is avirulent in mice (29, 30). To investigate if the reduced growth and deficient adherence of A. fumigatus Δ*sglA* contribute to the inability to establish infection, the virulence of the WT, Δ*sglA*, and Δ*sglA*::*sglA*^+^ strains in immunocompromised mice was tested. Interestingly, while all mice that were administered the WT or complemented strain succumbed to fatal infection at nearly identical rates, all mice that were intranasally (i.n.) inoculated with A. fumigatus Δ*sglA* conidia survived to the experimental endpoint, with no outwardly visible signs of morbidity (Fig. 3A). The organ burden was determined for the surviving A. fumigatus Δ*sglA*-inoculated mice, and the infection was found to be fully cleared from the lungs, with no extrapulmonary dissemination being observed (Fig. 3B). To confirm these data, histopathology was performed at day 30 for surviving Δ*sglA* strain-inoculated mice and compared to that of uninfected mice (Fig. 3C). Indeed, the lungs of uninfected mice very closely resembled the lungs of A. fumigatus Δ*sglA*-inoculated mice at the day 30 endpoint. These data collectively suggest that the Δ*sglA* mutant is avirulent and fully cleared from the lungs, with no prolonged inflammation, in a corticosteroid-immunosuppressed mouse model of infection.

### Immunization with A. fumigatus Δ*sglA* confers complete host protection against a lethal WT challenge.

**FIG 4:**
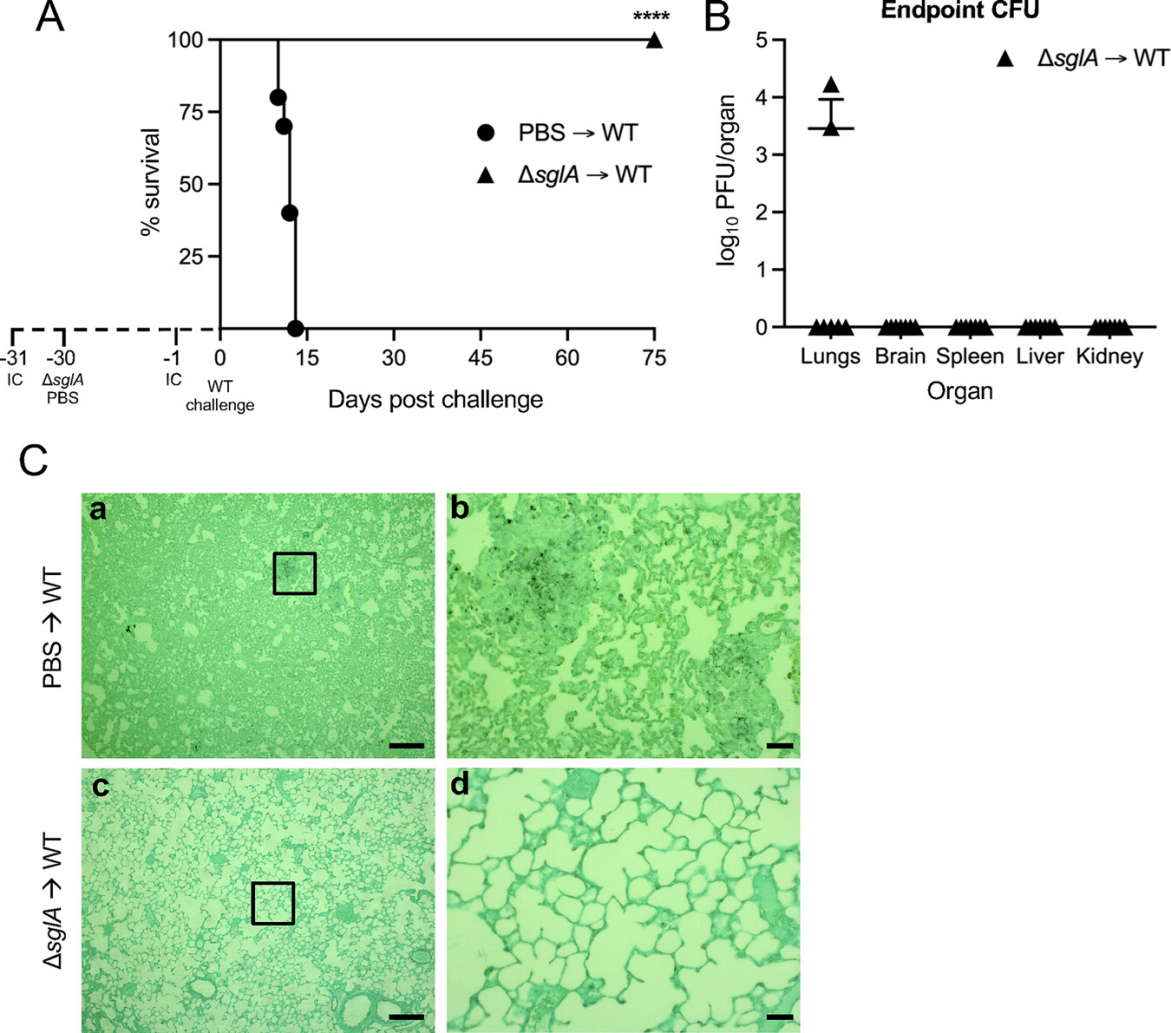
Immunization with A. fumigatus Δ*sglA* confers complete host protection against the WT strain. (A) CBA/J mice (*n* = 10 mice/group) were immunocompromised (IC) via the subcutaneous administration of 1 mg/mouse triamcinolone acetonide 24 h prior to the intranasal instillation of either A. fumigatus Δ*sglA* (vaccinated) or PBS (unvaccinated). All mice were infected with 2 × 10^4^ WT conidia 30 days later, with a second IC administration 24 h previously, and assessed for survival over 75 days. (B) Mice that survived the 75-day challenge phase were sacrificed, and the organ fungal burden was quantified (*n* = 7 mice/group). (C) Representative lung histopathology of an unvaccinated PBS-treated mouse and an Δ*sglA* strain-vaccinated mouse (*n* = 3 mice under each condition). Bars = 200 μm (a and c) and 50 μm (b and d). Graphed data represent the percent survival (A) and the means ± SD (B). Statistical significance was determined by the Mantel-Cox log rank test (A). Significance is indicated in panel A (****, *P < *0.001 for Δ*sglA* → WT versus PBS → WT).

Since A. fumigatus Δ*sglA* was found to be avirulent in mice and was eliminated by day 30, the protective nature of this mutant against a subsequent WT challenge was assessed. Because IA occurs mostly in immunocompromised individuals, the protective nature of the Δ*sglA* mutant was tested in two immunodeficient animal models of aspergillosis: the first group consisted of mice treated with a corticosteroid to induce phagocyte immunosuppression, and the second group of mice was treated with cyclophosphamide to induce leukopenia. Thus, corticosteroid-treated mice were intranasally immunized with A. fumigatus Δ*sglA* and subsequently challenged with the WT 30 days later. Indeed, all mice that previously received A. fumigatus Δ*sglA* fully survived until the experimental endpoint, while all PBS-treated mice succumbed to infection prior to day 15 (Fig. 4A). The organ fungal burden was examined in mice that survived until the experimental endpoint, and we found that 5 of the 7 mice had cleared the WT strain from the lungs, whereas all 7 mice had no extrapulmonary dissemination (Fig. 4B).

To confirm the results of the organ burden analysis, the lung histopathology of PBS-treated mice that succumbed to infection showed highly inflamed alveolar tissue with concentrated regions of Aspergillus conidia (Fig. 4Ca and b), while Δ*sglA* mutant-inoculated mice that survived the infection displayed healthy, uninflamed lung tissue with no fungal foci (Fig. 4Cc and d). These data ultimately indicate that the administration of A. fumigatus Δ*sglA* confers complete host protection against a lethal WT challenge and the resolution of alveolar tissue inflammation and suggest that the live, attenuated A. fumigatus Δ*sglA* mutant represents a viable vaccine candidate against aspergillosis in immunosuppressed mice.

### Immunization with heat-killed A. fumigatus Δ*sglA* conidia confers complete host protection.

**FIG 5:**
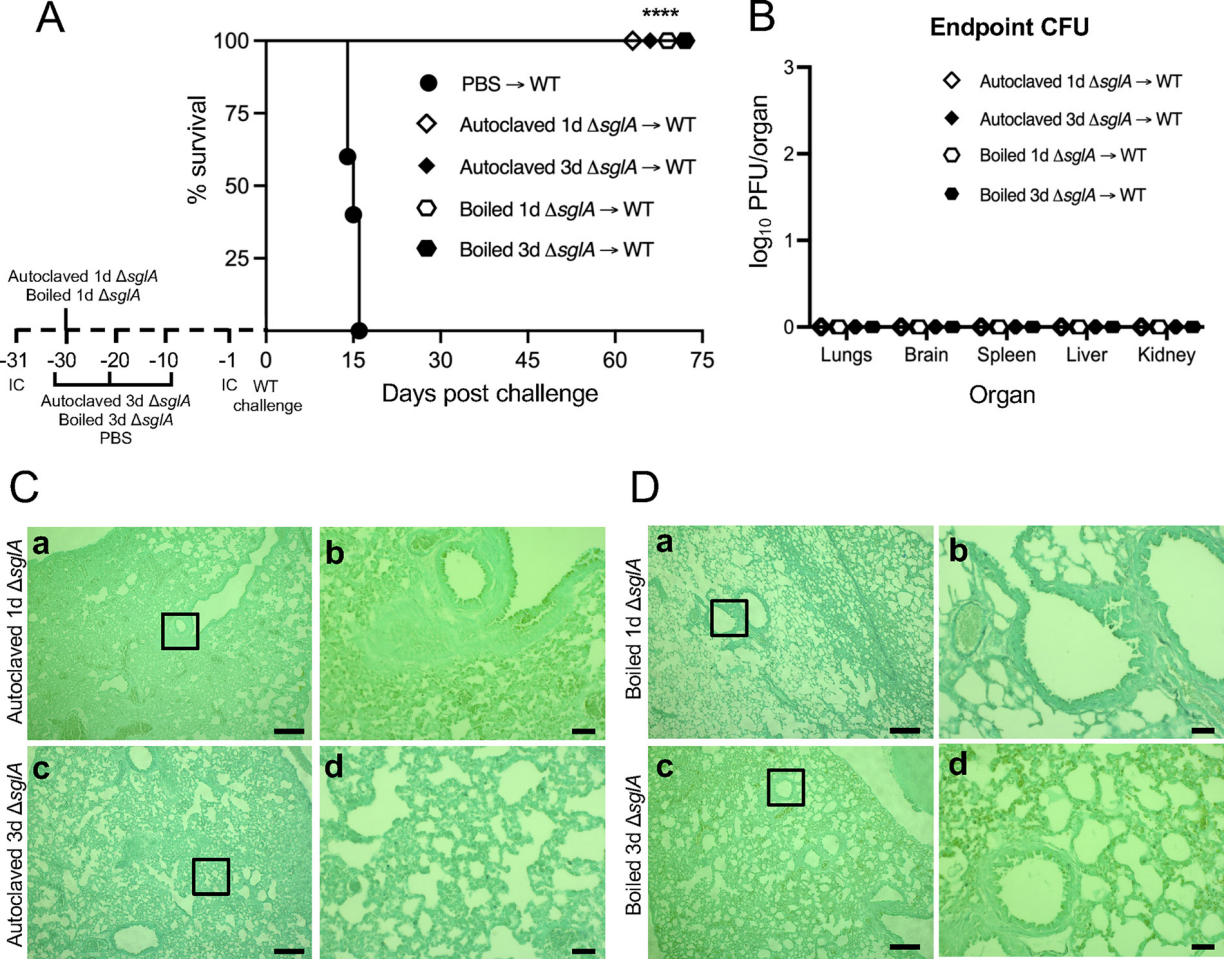
Immunization with autoclaved or boiled A. fumigatus Δ*sglA* conidia confers complete host protection. (A) CBA/J mice (*n* = 10 mice/group) were immunocompromised (IC) via the subcutaneous administration of 1 mg/mouse triamcinolone acetonide 24 h prior to the intranasal instillation of autoclaved or boiled A. fumigatus Δ*sglA* conidia (vaccinated) or PBS (unvaccinated). Mice received either 1 dose (autoclaved 1d and boiled 1d) 30 days prior to the WT challenge or 3 doses (autoclaved 3d and boiled 3d) 30, 20, and 10 days prior to the WT challenge. All mice were challenged with the lethal WT strain, with a second IC administration 24 h previously, and assessed for survival. (B) Mice that survived the challenge phase were sacrificed, and the organ fungal burden was quantified (*n* = 7 mice/group). (C and D) Representative lung histopathology for mice vaccinated with autoclaved (C) and boiled (D) Δ*sglA* conidia that survived until the experimental endpoint (*n* = 3 mice under each condition). Bars = 200 μm (Ca and c and Da and c) and 50 μm (Cb and d and Db and d). Graphed data represent the percent survival (A) and the means ± SD (B). Statistical significance was determined by the Mantel-Cox log rank test (A). Significance is indicated in panel A (****, *P < *0.001 for all surviving groups versus PBS → WT).

We recently reported that mice immunized with 2 doses of heat-killed (HK) C. neoformans Δ*sgl1* were completely protected from a lethal WT challenge (33). Because the administration of A. fumigatus Δ*sglA* conferred complete host protection against the WT strain, we then wanted to assess the efficacy of the HK A. fumigatus Δ*sglA* mutant in inducing host protection. To investigate this, we tested two HK preparations of A. fumigatus Δ*sglA*, autoclaved and boiled at 100°C for 30 min, both of which abolish conidial viability. Thus, immunocompromised mice were intranasally inoculated with either 1 dose of autoclaved or boiled HK A. fumigatus Δ*sglA* conidia 30 days prior to WT challenge or 3 doses of autoclaved or boiled HK A. fumigatus Δ*sglA* conidia on days −30, −20, and −10 prior to WT challenge. Strikingly, while all lethally challenged PBS-treated (unvaccinated) mice fully succumbed to the infection by day 16 postchallenge, all mice that received any iteration of HK A. fumigatus Δ*sglA* administration exhibited complete survival until the experimental endpoint (Fig. 5A). Both 1 dose and 3 doses of autoclaved or boiled immunizations fully protected immunocompromised mice from otherwise lethal infection, with 100% survival until day 75 postchallenge (Fig. 5A). Organ fungal burden analysis showed that all mice in each group fully cleared the WT strain from the lungs, with no extrapulmonary dissemination being observed (Fig. 5B).

The histology of the lungs from surviving mice that received the HK A. fumigatus Δ*sglA* vaccine iterations displayed an unequivocal resolution of infection (Fig. 5Ca to d and Fig. 5Da to d). No observable fungal cells were found in mice vaccinated with autoclaved HK A. fumigatus Δ*sglA* conidia with either 1 dose (Fig. 5Ca and b) or 3 doses (Fig. 5Cc and d) or in mice vaccinated with boiled HK A. fumigatus Δ*sglA* conidia with either 1 dose (Fig. 5Da and b) or 3 doses (Fig. 5Dc and d). Interestingly, the degrees of inflammation were different between the two groups: minimal lung inflammation was observed in mice administered 3 doses of autoclaved HK A. fumigatus Δ*sglA* conidia, which was similar to mice administered 1 dose of boiled HK A. fumigatus Δ*sglA* conidia, also displaying minimal lung inflammation. Overall, these data show that 1 dose of HK A. fumigatus Δ*sglA* conidia was sufficient to confer complete host protection in corticosteroid-treated mice and induce protective host immunity necessary to clear the WT strain from the lungs. These data also confirmed the results of previous studies in which an HK C. neoformans Δ*sgl1* strain completely protected immunocompromised mice from a lethal WT challenge (33), suggesting that an HK vaccine formulation enriched in SGs could be a valuable option for protecting individuals at risk of developing invasive cryptococcosis and aspergillosis.

### Vaccination with live or heat-killed A. fumigatus Δ*sglA* conidia protects neutropenic mice from developing invasive aspergillosis.

The most dominant risk factor for IA is neutropenia resulting from chemotherapy, certain types of cancer, or comorbidities. Whereas corticosteroid-induced immunosuppression eventually causes a hyperinflammatory host response in the lungs against noninvasive fungi, with tissue necrosis resulting from phagocyte loss of function, cytokine production, and attenuated effector cell recruitment (5, 6), the lungs of neutropenic mice show less inflammation with thrombosis and hemorrhage from extensive hyphal growth, leading to extrapulmonary dissemination (5, 6). Thus, we aimed to test the effect of our vaccines, both live and HK A. fumigatus Δ*sglA*, on this severe immunocompromised mouse model, which more closely mimics the most common human predisposing conditions for IA.

**FIG 6:**
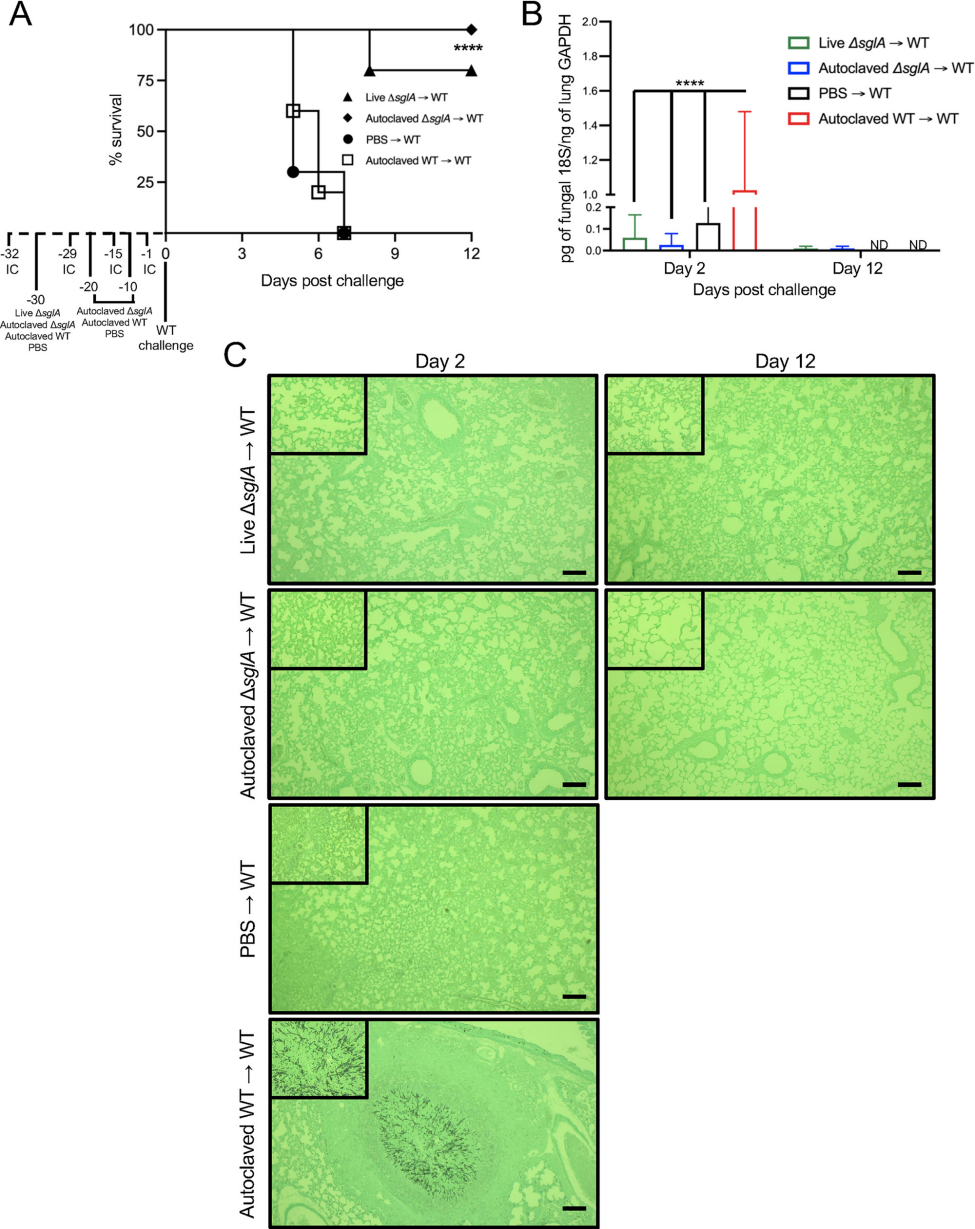
Immunization with either live or HK A. fumigatus Δ*sglA* conidia completely protects cyclophosphamide-induced leukopenic mice from lethal WT infection. (A) CBA/J mice (*n* = 10 mice/group) were immunocompromised with cyclophosphamide to induce neutropenia 2 days prior to the administration of live Δ*sglA* conidia 30 days prior to WT challenge or 3 doses of HK Δ*sglA* conidia, PBS, or HK WT conidia 30, 20, and 10 days prior to WT challenge and monitored for survival for 12 days postchallenge. Neutropenia was maintained with subsequent administrations of cyclophosphamide on days −29, −15, and −1. Significance was determined by the Mantel-Cox log rank test. Significance is indicated in panel A (****, *P < *0.001 for either live Δ*sglA* or HK Δ*sglA* conidia versus PBS → WT). (B) The lung fungal burden was assessed by qPCR determination of the concentration of fungal 18S DNA compared to the concentration of GAPDH from mouse lungs on days 2 and 12 after WT challenge. Graphed data represent the means ± SD of the fold changes in 18S fungal DNA/mouse lung GAPDH. Significance was determined via two-way ANOVA using Tukey’s multiple-comparison test for *P* value adjustment (****, *P < *0.001 for HK WT → WT compared to the other 3 groups). ND, not done for unvaccinated mice that did not survive until day 12 after WT challenge. (C) Lung histopathology was assessed for each group on days 2 and 12 postchallenge (*n* = 3 mice/group/day) to determine the progression of disease in unvaccinated mice (treated with PBS and HK WT conidia) and the resolution of infection in vaccinated mice (live Δ*sglA* and HK Δ*sglA* conidia). A representative image is shown for each group. Bars = 50 μm. The insets at the top left are magnified areas of interest from each field of view.

Mice were administered cyclophosphamide to induce leukopenia and then received either live Δ*sglA* conidia, HK Δ*sglA* conidia, PBS, or HK WT conidia, and 30 days later, they were lethally challenged with WT conidia. We found that all of the unvaccinated mice (PBS treated and HK WT treated) fully succumbed to fatal WT infection within 7 days (Fig. 6A). Amazingly, 100% of the mice vaccinated with HK Δ*sglA* conidia and 80% of the mice vaccinated with live Δ*sglA* conidia survived the WT challenge (Fig. 6A). Although we did not observe 100% protection in the mice vaccinated with live Δ*sglA* conidia, the median survival time for these mice was still significantly longer than that for either the PBS-treated or HK WT-treated mice.

To assess the level of lung colonization among the distinct groups, we quantified the fungal burdens over the course of the infection on days 2 and 12 after WT challenge using quantitative PCR (qPCR), which provides a better tool for the detection of mold burdens than plating of organ homogenates. We observed that neutropenic mice immunized with live Δ*sglA* or HK Δ*sglA* conidia had significantly lower levels of fungal 18S DNA 2 days after WT challenge than the mice administered HK WT conidia (Fig. 6B). Additionally, we also observed a modest, yet not significant, difference in fungal 18S DNA levels in the mice immunized with live Δ*sglA* or HK Δ*sglA* conidia compared to the PBS-treated mice. Finally, there was no quantifiable 18S fungal DNA in the mice immunized with live Δ*sglA* or HK Δ*sglA* conidia by day 12 after WT challenge, indicating that these mice not only survived the WT challenge but also were able to clear the WT conidia from the lungs.

To further confirm the lung burden analyzed by qPCR, we then analyzed lung histology for each of the groups at the same day 2 and day 12 time points (Fig. 6C). Similar to what was observed in the fungal burden experiments, neutropenic mice administered either live or HK A. fumigatus Δ*sglA* conidia exhibited healthy, uninflamed lung tissue on both days 2 and 12 postchallenge, with no observable fungal cells in the lungs (Fig. 6C). As expected, neutropenic mice administered either PBS or HK WT conidia displayed a significant level of inflammation in the lungs, and fungal cells were clearly seen to infiltrate the lung tissue. Interestingly, only mice receiving HK WT conidia displayed hypha formation (Fig. 6C), which was present in an isolated area surrounded by strong host hyperinflammation. In fact, all of these mice eventually succumbed to the infection. Overall, these data showed that even if the live, attenuated vaccine was able to provide protection, the HK vaccine, consisting of autoclaved Δ*sglA* conidia, was superior in conferring protection, even in a neutropenic mouse model of IA. Our results are encouraging and stimulate further studies for the research and development of a new and safe vaccine against IA and, possibly, other fungal diseases.

## DISCUSSION

In the present study, we have characterized the Δ*sglA* mutant of A. fumigatus engineered to accumulate SGs, representing a potential vaccine against IA. We found that the deletion of *sglA* caused a dramatic delay in hyphal differentiation (Fig. 1) and the production of a dense polysaccharide-rich ECM (Fig. 2A), resulting in attenuated adhesion capabilities and biofilm formation (Fig. 2B). From studies *in vivo*, we found that A. fumigatus Δ*sglA* was avirulent in two models of immunocompromised mice and was fully cleared from the lungs, with no lasting inflammation or tissue damage (Fig. 3 and 6). Importantly, both immunosuppressed and neutropenic mice previously inoculated with either live or HK Δ*sglA* conidia were fully protected from a subsequent lethal WT challenge (Fig. 4 to 6; see also Fig. S4 in the supplemental material), demonstrating the potential clinical application of our mutant, particularly the HK form, in individuals at risk of developing IA.

10.1128/mbio.02328-22.4 FIG S4 Immunization with either live or HK A. fumigatus Δ*sglA* conidia completely protects corticosteroid-treated mice from lethal WT infection. (A) CBA/J mice (*n* = 10 mice/group) were immunosuppressed with 1 mg/mouse triamcinolone acetamide 1 day prior to the administration of live Δ*sglA* conidia 30 days prior to WT challenge or 3 doses of HK Δ*sglA* conidia, PBS, or HK WT conidia 30, 20, and 10 days prior to WT challenge and monitored for survival for 30 days after challenge. Immunosuppression was continued every 10 days after the first dose until the experimental endpoint. Significance was determined by the Mantel-Cox log rank test. Significance is indicated in panel A (****, *P < *0.001 for either live Δ*sglA* or HK Δ*sglA* conidia versus PBS → WT). (B) Lung histopathology was assessed for each group on days 4, 10, and 21 after challenge (*n* = 3 mice/group/day) to determine the progression of disease in unvaccinated mice (treated with PBS and HK WT conidia) and the resolution of infection in vaccinated mice (live Δ*sglA* and HK Δ*sglA* conidia). A representative image is shown for each group. Bars = 50 μm. The insets at the top left are magnified areas of interest from each field of view. Download FIG S4, TIF file, 1.3 MB. Copyright © 2022 Fernandes et al. 2022 Fernandes et al. https://creativecommons.org/licenses/by/4.0/ This content is distributed under the terms of the Creative Commons Attribution 4.0 International license.

A. fumigatus Δ*sglA* was found to be avirulent *in vivo* in immunocompromised mice, resulting in complete host survival, the clearance of the mutant from the lungs, and the resolution of inflammation after clearance (Fig. 3 and 6 and Fig. S4). The avirulent phenotype may be attributed in part to the delayed hyphal growth (Fig. 1) and the reduced capacity for biofilm formation (Fig. 2) observed *in vitro* in A. fumigatus Δ*sglA*. Normally, resting conidia begin to swell upon inhalation into the lower airways, shedding the hydrophobic RodA layer and becoming metabolically active (43). Unlike immunocompetent hosts, swollen conidia are not cleared by immunocompromised hosts, resulting in invasive hyphal growth in neutropenic hosts or a hyperinflammatory response during corticosteroid-induced immunosuppression (6). However, the Δ*sglA* mutant remains as conidia for extended periods, possibly allowing the host to recognize and respond to the inhaled conidia, even if the host is immunocompromised. It is possible that the hydrophilic extracellular polysaccharide layer observed in A. fumigatus Δ*sglA* can account for the increased exposure to the antigen(s), perpetuating augmented host recognition since the ECM observed in the mutant strain is different from the bona fide architecture and sugar composition found in the WT strain (Fig. 2A). The exposure and presentation of these Aspergillus antigens are now able to stimulate a protective host response against primary and, eventually, subsequent lethal WT challenge. It is interesting that upon the clearance of the mutant, the host is able to resolve lung inflammation, with minimal, if any, tissue damage. We therefore hypothesize that the delayed conidium-to-hypha transition, the altered presentation of cell surface antigens, or a combination of both of these facets stimulates the host to positively respond to A. fumigatus Δ*sglA*.

Early host recognition and responses to inhaled conidia are carried out chiefly by innate resident alveolar macrophages, dendritic cells, and lung epithelial cells (15, 16, 43). Immunocompetent hosts properly handle swelling conidia, suppressing the progression toward hyphal transition. However, immunocompromised hosts, upon corticosteroid treatment, exhibit disrupted phagocyte uptake, a lack of Aspergillus killing, and aberrant cytokine production, resulting in a hyperinflammatory response in the lungs that leads to tissue damage (5, 6, 44). It is noteworthy to mention that we induced immunosuppression via corticosteroid or cyclophosphamide administration prior to the administration of A. fumigatus Δ*sglA*. This suggests that host immunity necessary to recognize and respond to the mutant and eventually carry out host protection against WT challenge is not lost under either of these two conditions of immunodeficiency.

The dense ECM in A. fumigatus Δ*sglA*, which was shown to be composed mainly of glycoconjugate antigens possibly engulfed within sugar-coated SGs, may stimulate advantageous pattern recognition receptors (PRRs) on innate immune cells or lung epithelial cells. In fact, protective host immunity against A. fumigatus requires several host cell PRRs, most notably dectin-1 and Toll-like receptor 2 (TLR2), aiding in the shift toward a host-protective Th1 response (10, 45–47). Although there are no host PRRs that have been identified to date that recognize fungal SGs, we hypothesize that the accumulation of SGs may facilitate and activate an antifungal host immune response via PRR ligation. Future investigation into host PRR ligation of fungal SGs is warranted.

Complete host protection during corticosteroid-induced immunosuppression with A. fumigatus Δ*sglA* was observed with the live, attenuated mutant and also with HK (autoclaved or boiled) conidia (Fig. 4 and 5 and Fig. S4). Protection with autoclaved or boiled conidia is of greater clinical interest since vaccination with a live, attenuated mutant poses potential health safety concerns for individuals at risk of IA due to the fact that a live, attenuated strain is still a live fungus. But this should not be a problem because only one administration of either the autoclaved or boiled Δ*sglA* conidia was sufficient for complete protection of the host. This was not observed in studies using HK C. neoformans Δ*sgl1* conidia, where 2 doses of HK conidia were necessary to obtain 100% protection, while 1 dose conferred 70% protection in immunodeficient mice (33).

Considering the reduced inflammatory response in the lung (Fig. 5C), we opted to use 3 doses of the autoclaved formulation in the neutropenic model, and this dose was extremely effective in protecting the host (Fig. 6A) and superior to immunization with live Δ*sglA* conidia. This is most likely due to the fact that repeated exposures to HK Δ*sglA* conidia over the 30-day period may build up a stronger adaptive immune defense upon WT challenge. This will require a comprehensive immunological analysis of the host immune cell types necessary to induce this protection.

As mentioned above, both the autoclaved and boiled Δ*sglA* conidia provided complete protection against WT infection. Remarkably, the lung histology differed depending on the administration schedule. Mice given 3 doses of autoclaved A. fumigatus Δ*sglA* conidia displayed some lung inflammation although minimal (Fig. 5Cc and d), while mice given 1 dose of boiled A. fumigatus Δ*sglA* conidia displayed a total lack of lung inflammation (Fig. 5Da and b). It is likely that repeated exposure to the autoclaved mutant cells induces stronger host immunity, particularly on T cells (48). Canonical αβ T cells need antigen-presenting cells (APCs) to prime their protective differentiation states, so an increased presentation of antigens (3 doses of autoclaved conidia) would be sufficient for increased memory T cell populations. On the other hand, boiled mutant cells may stimulate sufficient host immunity after only 1 administration, and additional administrations prior to WT challenge result in a sublethal yet hyperinflammatory host response, as observed via lung histology (Fig. 5Dc and d). Since boiling A. fumigatus Δ*sglA* conidia is less severe than autoclaving the mutant conidia, 1 dose of boiled Δ*sglA* conidia may provide ample antigen exposure to stimulate a protective memory T cell population in the immunization phase of the infection. Overall, we clearly show here that both 3 doses of autoclaved and 1 dose of boiled conidia provided complete immunity to WT infection, with the least inflammation after challenge.

We have previously shown that vaccination with C. neoformans Δ*sgl1* was characterized by the recruitment of canonical CD4^+^ and/or CD8^+^ T cells, noncanonical γδ T cells, and neutrophils to the lungs, inducing a proinflammatory, host-protective immune response yet also displaying an obvious resolution of pulmonary tissue inflammation (29). Because we are assessing the same fungally derived SGs that accumulate in both C. neoformans Δ*sgl1* and A. fumigatus Δ*sglA*, the inflammatory effector leukocyte infiltrates in the lungs can be predicted to be similar. The optimal immunization scheme will need to be further assessed by investigations into the type of T cell immunity (17, 49) stimulated by our mutant as well as other effector cells recruited by WT challenge, a necessary step for a better understanding of the immune mechanisms by which A. fumigatus Δ*sglA* confers host protection against lethal IA. Nevertheless, there will be obvious differences since we have shown that neutrophils are not a mandatory effector leukocyte population in the present study, although they were necessary for host protection in C. neoformans Δ*sgl1* vaccination.

Our present work uncovers a novel aspect of host immunity to A. fumigatus since this is the first HK Aspergillus mutant that fully protects mice from a lethal WT challenge in two models of immunodeficiency. Our observations suggest that SG accumulation in the Δ*sglA* mutant facilitates the exposure of fungal antigens to immune cells, promoting fungal clearance after a subsequent WT infection. More importantly, the fungal antigen necessary for mounting a protective host immune response does not seem to be lost, even after autoclaving the mutant conidia. Cenci and colleagues immunized mice with either live, HK, or crude filtrates of A. fumigatus and reported that protective host responses were observed for the live and crude filtrates but not the HK filtrate (50). In addition, mice were immunocompromised after the vaccine administrations, and the protection observed for the live and crude filtrates required CD4^+^ T cells, ultimately deterring any clinical potential. Notwithstanding, it is worthwhile to mention work from two other groups that used HK fungi other than Aspergillus to confer protection against lethal Aspergillus infection. Clemons and colleagues immunized mice with HK Saccharomyces cerevisiae and observed prolonged survival, although all mice still succumbed to fatal WT infection (51). In addition, Wang and colleagues used an HK C. neoformans mutant to fully protect mice from a lethal A. fumigatus challenge, although these mice were vaccinated prior to being immunosuppressed with cyclophosphamide (28). Overall, our vaccination strategy using HK A. fumigatus Δ*sglA* conidia administered prior to the induction of any immunodeficiency offers novel insight into future potential vaccination strategies against IA in both neutropenic and immunosuppressed hosts.

Intriguingly, SGs alone were not found to induce host immunity to C. neoformans. Complete host protection against WT infection required the glucuronoxylomannan (GXM) capsule of C. neoformans in conjunction with the accumulated SGs, since an acapsular mutant accumulating SGs (C. neoformans Δ*cap59* Δ*sgl1*) did not confer protection (32). Whether A. fumigatus Δ*sglA* would require an additional fungal component to confer protection remains to be elucidated. Based on our observations (Fig. 2), future investigations will focus on specific components of the conidial surface, such as exposed cell wall components, including galactosaminogalactans, *N*-acetylglucosamine, and chitin, since these were the three differentially expressed components compared to the WT strain. It will be interesting to investigate whether A. fumigatus cells accumulating SGs but lacking some of these sugar moieties would lose their host-protective properties, similar to what we reported previously for C. neoformans Δ*cap59* Δ*sgl1* (32).

In summary, we have presented an innovative study producing A. fumigatus Δ*sglA* as a potential vaccine candidate for protection against IA in either neutropenic or corticosteroid-treated hosts. To our knowledge, this is the first report of an HK mutant strain of Aspergillus that induces full prophylactic protection against a lethal WT challenge, using mice that were immunocompromised prior to the administration of the vaccine formulations. This is the second opportunistic fungal pathogen that we have engineered to accumulate SGs that provided full immunity against a subsequent lethal infection, highlighting the exciting potential of fungal SGs as immunostimulating adjuvant compounds for the research and development of a new fungal vaccine.

## MATERIALS AND METHODS

### Strains and culture conditions.

The A. fumigatus strains used in this work are listed in Table S2 in the supplemental material. Strains were grown at 37°C in either complete medium (yeast extract-glucose [YG] [2% {wt/vol} glucose, 0.5% {wt/vol} yeast extract, 1× trace elements]) or minimal medium (MM) (1% [wt/vol] glucose, 1× high-nitrate salts, 1× trace elements [pH 6.5]). High-nitrate salts and the trace element solution were prepared as previously described (37). For solid YG (YAG) medium and MM, 2% (wt/vol) agar was added. Δ*sglA* and Δ*sglA*::*sglA*^+^ transformants were selected by plating the protoplasts in modified YAG medium containing 1% (wt/vol) agar, 0.6 M KCl, and 1.2 M sorbitol. When required, the medium was supplemented with 1.2 g/L of uracil and uridine, generating YUU medium or MM+UU.

10.1128/mbio.02328-22.6 TABLE S2 A. fumigatus strains used in this study. Download Table S2, TIF file, 0.01 MB. Copyright © 2022 Fernandes et al. 2022 Fernandes et al. https://creativecommons.org/licenses/by/4.0/ This content is distributed under the terms of the Creative Commons Attribution 4.0 International license.

### Construction of A. fumigatus Δ*sglA* and Δ*sglA*::*sglA*^+^.

An *sglA* knockout cassette containing 2 kb of the 5′ and 3′ untranslated regions (UTRs) flanking the *sglA* ORF (Afua_3g08820) was generated in order to facilitate homologous recombination. The *pyrG* gene was selected as a prototrophy marker. The resulting cassette, illustrated in Fig. S1, was synthesized by Biobasic Inc. and amplified using primers 5′ UTR fw and 3′ UTR rv (Table S3). The deletion construct was inserted into ΔKu80 *pyrG1* protoplasts as previously described (37), and transformants were selected for their ability to grow in the absence of uracil and uridine.

10.1128/mbio.02328-22.7 TABLE S3 Primers used in this study to amplify the deletion and reconstitution cassettes (5′ UTR fw and 3′ UTR rv), the 5′-UTR sequence used as a probe for Southern blot analysis (Southern blot probe fw and Southern blot probe rv), and fungal 18S rRNA (18S fw and 18S rv) and mouse GAPDH (GAPDH fw and GAPDH rv) sequences for qPCR analysis. Download Table S3, TIF file, 0.01 MB. Copyright © 2022 Fernandes et al. 2022 Fernandes et al. https://creativecommons.org/licenses/by/4.0/ This content is distributed under the terms of the Creative Commons Attribution 4.0 International license.

The reconstitution cassette used to generate the Δ*sglA*::*sglA*^+^ strain consisted of the *sglA* gene, the *hph* promoter, and coding sequences flanked by the 5′ and 3′ UTRs of *sglA* (Fig. S1). This cassette was synthesized and amplified with primers 5′ UTR fw and 3′ UTR rv (Table S3). All of the PCR amplifications were performed using TaKaRa Ex *Taq* DNA polymerase (TaKaRa Bio). The cassette was transformed into Δ*sglA* protoplasts, and its integration in the genomic DNA was initially verified by growing the colonies in the presence of 200 μg/mL of hygromycin B (38).

### DNA extraction and Southern blot analysis.

The integration of the deletion and reconstitution cassettes into the *sglA* locus was investigated by Southern blot analysis. WT, Δ*sglA*, and Δ*sglA*::*sglA*^+^ conidia were grown in 10 mL of YUU medium at 37°C overnight with agitation. DNA was isolated as described previously by Malavazi and Goldman (37), with a few modifications. Mycelium disruption was achieved by lyophilizing frozen suspensions instead of grinding them with a mortar and pestle. The 5′-UTR sequence used as a probe was amplified from the genomic DNA of ΔKu80 *pyrG*^−^ with the Southern blot 5′ fw and rv primers (Table S3). HindIII-digested DNA fragments were run on a 1% agarose gel overnight at 15 V and transferred to a nylon membrane as described previously by Singh et al. (52). Radioactive labeling of the probe with [α-^32^P]dCTP (Perkin-Elmer) was performed using a Random Primers DNA labeling system kit (Invitrogen) according to the manufacturer’s instructions. The membrane was exposed to Carestream BioMax MR film (Millipore-Sigma), and bands were developed using the SRX-101A medical film processor (Konica Minolta).

### Lipid extraction, SG analysis, and thin-layer chromatography.

A total of 1 × 10^6^ conidia of the WT, Δ*sglA*, and Δ*sglA*::*sglA*^+^ strains were inoculated into 10 mL of MM+UU and grown at 37°C with agitation. For SG quantification by liquid chromatography (LC)-MS, conidia were harvested straight from the solid plate (0 h) or grown for 12, 24, or 48 h, while the cultures used for thin-layer chromatography (TLC) were grown for 48 h. The suspensions were washed with sterile water and suspended in Mandala buffer (53). Lipid extraction was carried out as described previously by Singh and Del Poeta (54), with a few modifications. To facilitate the disruption of mycelia, samples were vortexed and sonicated for 2 min in the presence of 0.2-mm glass beads. The supernatant was collected, dried, and submitted to extraction by the Bligh-Dyer method (55). One-third of each sample obtained after Bligh-Dyer extraction was reserved for inorganic phosphate (P_i_) determination, while the remaining sample was subjected to alkaline hydrolysis of phospholipids (56). The SG concentration determined by mass spectrometry was further normalized to the P_i_ abundance (32). For TLC analysis, lipid extracts were suspended in 50 μL of chloroform-methanol (2:1) and applied onto a silica gel plate (Millipore-Sigma). Twenty micrograms of a sterylglucoside standard (Matreya) was included as a migration control. Samples were run in chloroform-methanol-water (65:25:4, vol/vol/vol) and stained with iodine.

### Microscopy.

To evaluate hyphal growth, 1 × 10^5^ conidia were inoculated onto a glass-bottom dish containing 2 mL of MM+UU and grown at 37°C for 6, 12, or 24 h. The fungal suspensions were analyzed by differential interference contrast (DIC) microscopy, and images were taken with the 40× objective of a Zeiss Observer D.1 microscope. The size of the hyphae was measured using ZEN software.

### Scanning electron microscopy of A. fumigatus.

A total of 1 × 10^6^ conidia of the WT, Δ*sglA*, and Δ*sglA*::*sglA*^+^ strains were statically grown in 10 mL of MM+UU at 37°C for 12 h. Suspensions were centrifuged and fixed for 2 h in 3% electron microscopy (EM)-grade glutaraldehyde diluted in 0.1 M sodium cacodylate buffer (pH 7). Samples were washed twice with 0.2 M sodium cacodylate buffer and dehydrated for 15 min with increasing concentrations of ethanol (50%, 70%, 90%, and 100%). Next, samples were submersed in hexamethyldisilazane (HMDS)-ethanol (1:2, 1:1, and 2:1) and pure HMDS solutions for 15 min and dried overnight at room temperature. Prior to scanning electron microscopy (SEM) characterization, samples were coated with Au-Pd (80:20) at a 6-nm thickness to allow electrons to dissipate from the surface. A scanning electron microscope (X-beam 340; Zeiss) was then used to characterize the morphology of the samples at 1 kV.

### Scanning electron microscopy of C. neoformans.

C. neoformans cells were grown as described previously (32), washed three times in PBS, and fixed in 2.5% glutaraldehyde type 1 (Electron Microscopy Sciences) diluted in 0.1 M sodium cacodylate buffer for 40 min at room temperature. Next, cells were washed in 0.1 M sodium cacodylate buffer containing 0.2 M sucrose and 2 mM MgCl_2_ and adhered to coverslips coated with 0.01% poly-l-lysine (Millipore-Sigma) for 20 min. Adhered cells were dehydrated in a series of freshly made solutions of graded ethanol (30, 50 and 70% for 5 min/step; 95% for 10 min and then 100% twice for 10 min/step). Samples were then subjected to critical-point drying (EM CPD 300; Leica) immediately after dehydration, mounted onto metallic stubs, coated with a 10- to 15-nm Au-Pd layer (Union FL-9496; Balzers), and visualized using a scanning electron microscope (Evo LS; Carl Zeiss) operating at 15 kV.

### Biofilm formation assay.

The quantification of the initial stages of biofilm formation in A. fumigatus was performed as described previously by Gravelat et al. (57). In brief, 10^5^ conidia were inoculated into 200 μL of MM+UU in 96-well plates and allowed to grow for 12 and 24 h at 37°C. Following incubation, the medium was removed, and the adhered mycelia were washed three times with sterile PBS. One hundred fifty microliters of a 0.5% (wt/vol) crystal violet solution was added to each well for 5 min to stain the residual mycelia. The excess stain was gently removed by washing once with sterile water. The residual biofilm was destained with 200 μL of 95% ethanol per well for 16 h at room temperature. The biofilm density was measured by determining the absorbance of the destaining solution at 570 nm. The results were normalized to the fluorescence values of resazurin (Millipore-Sigma), which was used at a concentration of 10% in a separate and independent experiment to evaluate the growth of the strains, and expressed as percent adherence.

### Detection of cell surface carbohydrates.

The detection of cell surface carbohydrates was performed as described previously by Manfiolli et al. (41), with a few modifications. Briefly, 10^3^ conidia of A. fumigatus strains were grown in 200 μL of MM+UU for 16 h at 37°C. The culture medium was removed, and the germlings were UV irradiated (600,000 μJ) and washed with PBS. For chitin staining, 100 μL of a PBS solution containing 10 μg/mL of CFW was added to UV-irradiated germlings, the germlings were incubated for 5 min at room temperature and washed three times with PBS, and the fluorescence was read at a 380-nm excitation wavelength and a 450-nm emission wavelength. For the other lectins, 200 μL of blocking solution (2% [wt/vol] fetal bovine serum, 1% [wt/vol] bovine serum albumin [BSA], 0.1% [vol/vol] Triton X-100, 0.05% [vol/vol] Tween 20, 0.05% [vol/vol] sodium azide, and 0.01 M PBS) was added, and the mixture was incubated for 30 min at room temperature. For *N*-acetylgalactosamine (GalNAc), *N*-acetylglucosamine (GlcNAc), or α-mannose staining, 200 μL of PBS with 0.1 mg/mL of SBA-FITC (Glycine max soybean lectin; Bioworld), WGA-FITC (lectin from Triticum vulgaris [wheat]; Millipore-Sigma), or ConA (concanavalin A)-FITC (Millipore-Sigma), respectively, was added to UV-irradiated germlings; the germlings were incubated for 3 h at room temperature and washed three times with PBS; and the fluorescence was read at a 492-nm excitation wavelength and a 517-nm emission wavelength. For β-glucan staining, 0.2 μg/mL of monoclonal anti-β-glucan antibody (Biosuppliers) was added to the germlings, and the mixture was incubated overnight at 4°C, followed by the addition of FITC-conjugated anti-mouse IgG antibody at a 1:1,000 dilution in PBS, which was incubated for 2 h at room temperature. Germlings were washed three times with PBS, and the fluorescence was read at a 492-nm excitation wavelength and a 517-nm emission wavelength. All of the experiments were performed with at least six repetitions, and the results were read on a SpectraMax i3 instrument (Molecular Devices). Normalization was done by parallel growth in the presence of resazurin (560-nm excitation and 590-nm emission).

### Study approval.

All animal procedures were approved by the Stony Brook University Institutional Animal Care and Use Committee (protocol number 341588) and were performed according to the guidelines of the American Veterinary Medical Association.

### *In vivo* immunosuppression.

Mice were immunosuppressed with the corticosteroid triamcinolone acetonide (stock number J63548; Alfa Aesar) as previously described (58, 59). For primary-infection studies, mice were immunosuppressed via the subcutaneous administration of 1 mg/mouse triamcinolone acetonide 1 day prior to the i.n. instillation of A. fumigatus WT, Δ*sglA*, or Δ*sglA*::*sglA*^+^ conidia. For vaccination studies, mice were treated with 1 mg/mouse triamcinolone acetonide 1 day prior to immunization with A. fumigatus Δ*sglA* conidia and again every 10 days to maintain immunosuppression.

Leukopenia was induced in mice using cyclophosphamide (Sigma-Aldrich). For vaccination studies, mice were intraperitoneally (i.p.) administered 150 mg/kg of body weight of cyclophosphamide 2 days prior to immunization. Neutropenia was then maintained for the entirety of the experiment, with subsequent i.p. administrations of 75 mg/kg of body weight of cyclophosphamide on days −29, −15, and −1. To protect mice from possible bacterial infection, the drinking water was supplemented with tetracycline (Sigma-Aldrich) to a final concentration of 200 mg/L, which was maintained for the entirety of the experiment.

### *In vivo* infection and survival studies.

Male and female CBA/J mice were purchased from Envigo. All animals had access to food and water *ad libitum*. All mice were allowed 1 week to acclimate upon arrival before any procedures began. For primary infections, 4- to 5-week-old mice were immunocompromised as described above. Twenty-four hours later, mice were i.p. anesthetized with a ketamine-xylazine solution (95 mg of ketamine and 5 mg of xylazine per kg of animal body weight). Anesthetized mice were i.n. injected with 2 × 10^4^ WT, Δ*sglA*, or Δ*sglA*::*sglA*^+^ conidia in 20 μL of PBS and monitored daily for survival until the predetermined experimental endpoints. Any mouse that exhibited labored breathing, had trouble getting to food or water, or had lost more than 20% of its body weight was euthanized. Mice that survived until the endpoint were euthanized by CO_2_ inhalation and used for organ fungal burden determinations or histopathology.

For vaccination studies, 4- to 5-week-old mice were immunocompromised 24 h prior to immunization with either 2 × 10^4^ live Δ*sglA* or 2 × 10^6^ autoclaved or boiled Δ*sglA* conidia in 20 μL of PBS. Autoclaved conidia were autoclaved to 121°C for 15 min, while boiled conidia were incubated at 100°C for 30 min. Conidial suspensions were cooled at room temperature prior to i.n. instillation. The viability of live and HK suspensions was monitored by plating onto YAG medium. Mock-immunized mice were given 20 μL of sterile PBS. Mice were lethally challenged with 2 × 10^4^ WT strain conidia at 30 days postimmunization. For immunization with HK Δ*sglA* conidia, mice were given either 1 dose (autoclaved or boiled) 30 days prior to WT infection or 3 doses (autoclaved or boiled) 30, 20, and 10 days prior to WT infection. All mice were monitored daily during the experiment and euthanized if signs of morbidity were observed. Mice that survived until the endpoint were euthanized via CO_2_ inhalation and used for organ fungal burden determinations or histopathology.

All statistical analyses were performed using GraphPad Prism 9 software. The sample sizes, statistical analyses, and statistical significance values are described in each figure legend. A *P* value of <0.05 was considered significant. No data were excluded from the figures or calculations of statistical significance.

### Organ fungal burden quantification.

Aspergillus colonization of distinct organs was assessed by the determination of the log_10_ CFU per milliliter or the amplification of fungal 18S DNA by qPCR. The lungs, brain, spleen, liver, and kidneys of euthanized mice were aseptically removed, and each organ was homogenized in 10 mL of sterile PBS using a Stomacher 80 blender for 2 min. Serial dilutions were plated onto YAG plates, and fungi were grown for 2 to 3 days at 37°C before being counted.

For qPCR analysis, the lungs were removed from euthanized mice and immediately frozen in liquid nitrogen. Organ processing was carried out as previously described (60). Briefly, the lungs were lyophilized for 2 to 3 days and ground to a powder using 2-mm glass beads. Next, samples were vigorously vortexed in lysis buffer and phenol-chloroform-isoamyl alcohol (25:24:1). The aqueous phase was collected, and the genomic DNA was precipitated with isopropanol. Fungal 18S and mouse glyceraldehyde-3-phosphate dehydrogenase (GAPDH) were amplified using SYBR green PCR master mix (Thermo Fisher Scientific), and the primers used are listed in Table S3.

### Histopathology.

For histological analysis, mice were euthanized at the experimental endpoint or upon the onset of invasive aspergillosis via CO_2_ inhalation. Lungs were aseptically removed, washed in PBS, fixed in a 10% formalin solution for 24 h, and dehydrated in ethanol. Dehydrated samples were infiltrated with paraffin, cut into 5-μm-thick slices, and stained with Grocott methenamine silver (GMS). Three lungs/group/time point were microscopically analyzed, and a representative image is shown for each group.
