# Supplementary figures and images for: Vaccination with Live or Heat-Killed Aspergillus fumigatus ΔsglA Conidia Fully Protects Immunocompromised Mice from Invasive Aspergillosis
Source: mBio. 2022 Sep 6;13(5):e02328-22. doi: 10.1128/mbio.02328-22 (PMC9600187; doi:10.1128/mbio.02328-22)

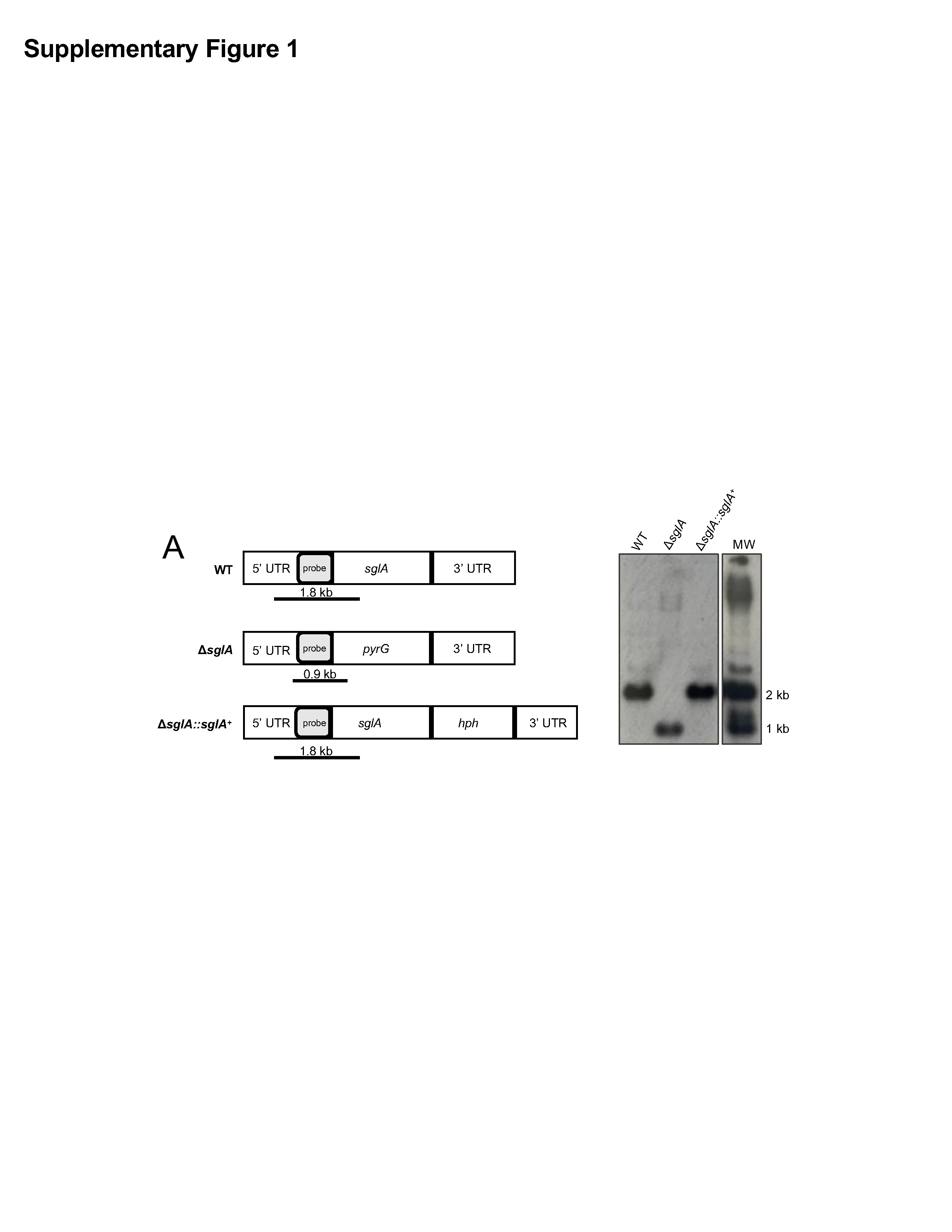

Supplement: FIG S1 [file mbio.02328-22-s0004.tif]

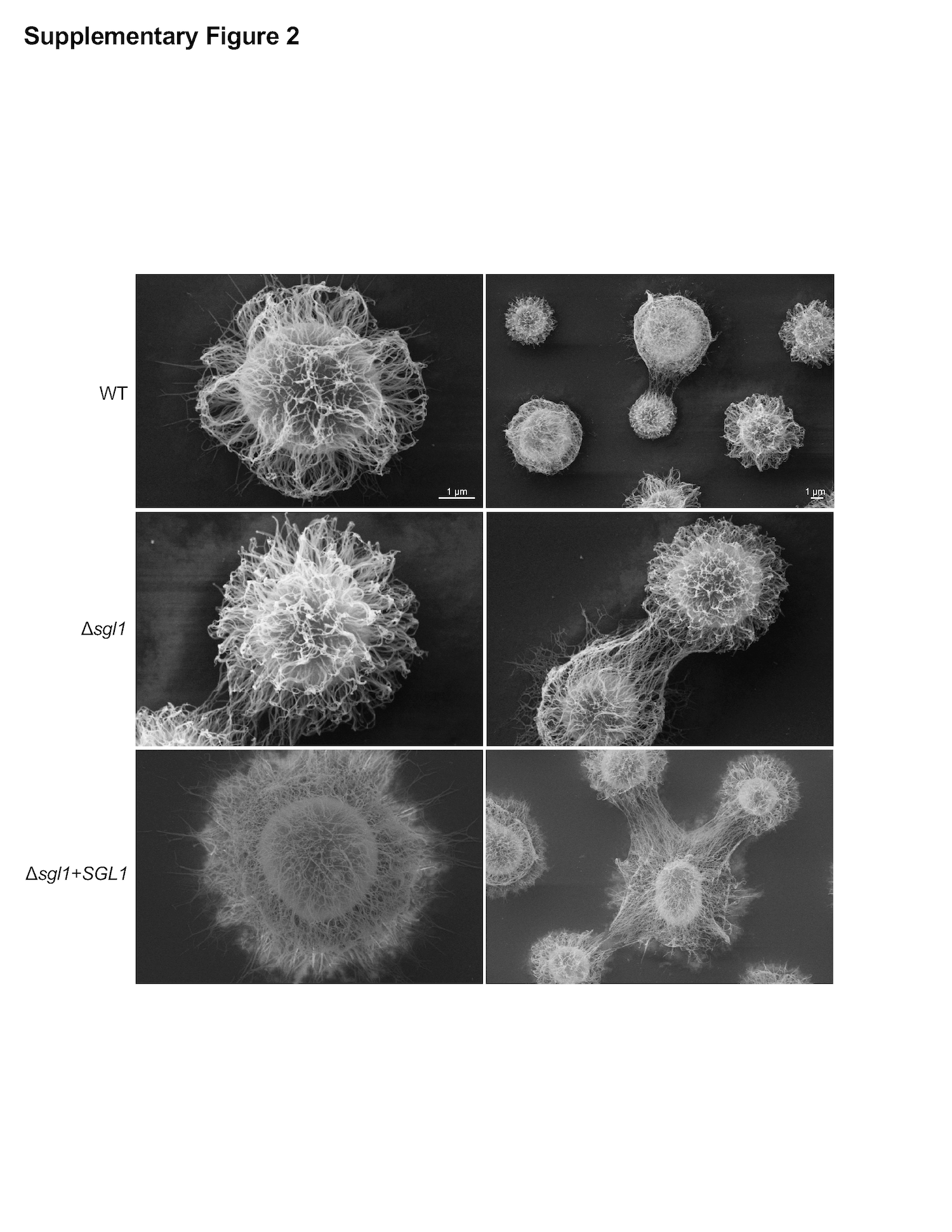

Supplement: FIG S2 [file mbio.02328-22-s0005.tif]

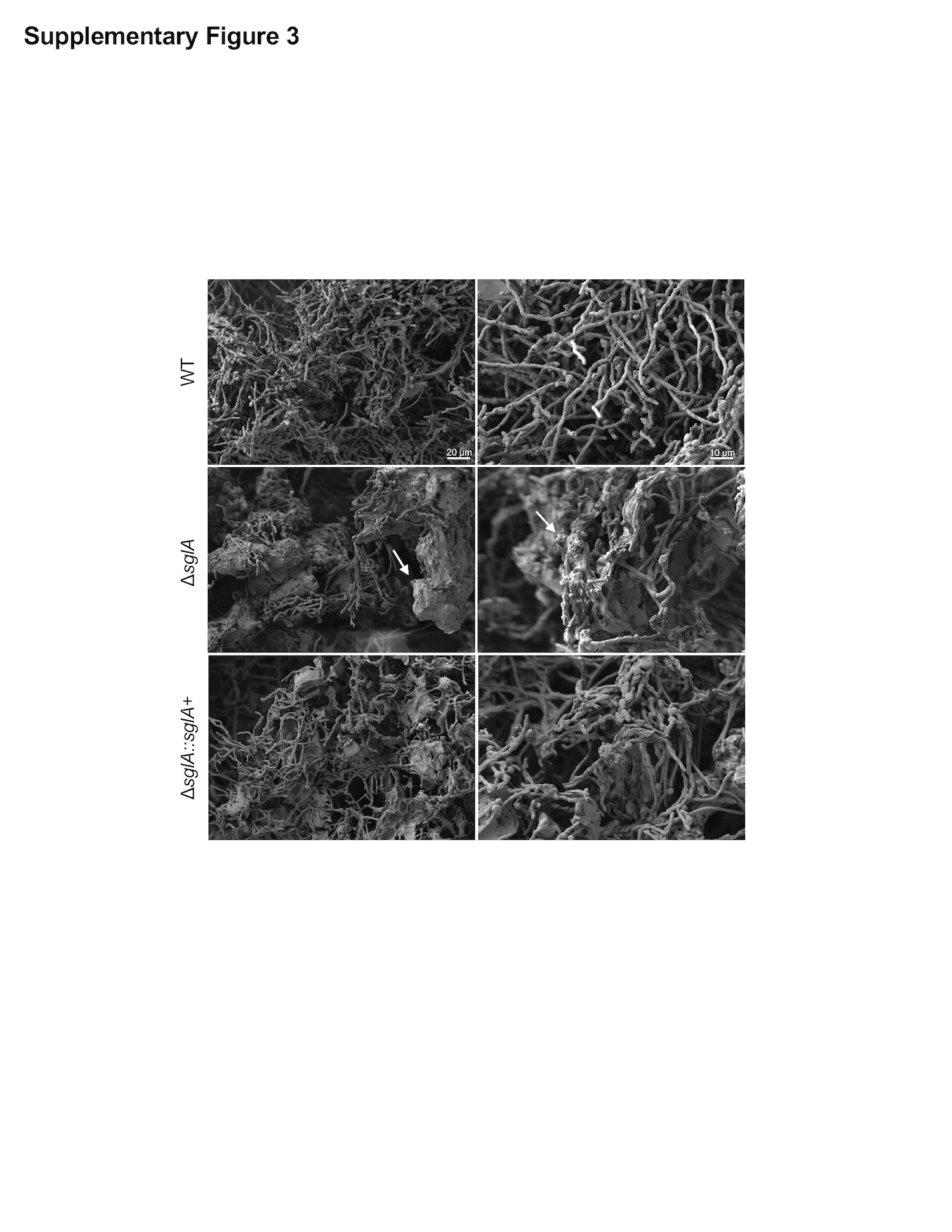

Supplement: FIG S3 [file mbio.02328-22-s0006.tif]

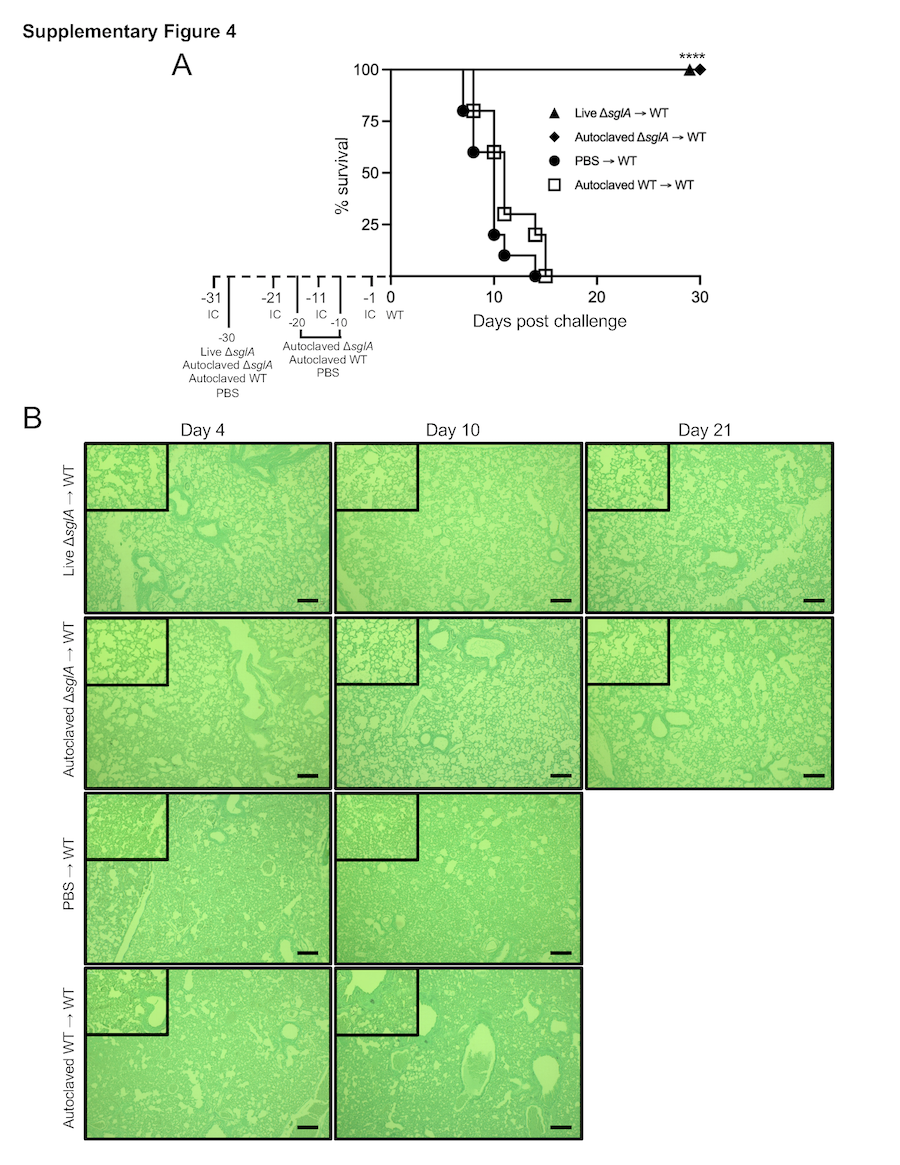

Supplement: FIG S4 [file mbio.02328-22-s0007.tif]
